# Identification and functional analysis of ULP2 SUMO protease mutants of the stress-tolerant yeast *Kluyveromyces marxianus*

**DOI:** 10.17912/micropub.biology.001816

**Published:** 2025-09-23

**Authors:** Catherine Hutson, Samuel Li, Keegan Sweeney, Oliver Kerscher

**Affiliations:** 1 Biology, William & Mary

## Abstract

*K. marxianus* ( *Km* ), a stress-tolerant yeast, is closely related to *S. cerevisiae* ( *Sc* ). Unlike *Sc* , *Km* yeast grows robustly in the presence of proteotoxic stressors, including temperatures up to 45°C and oxidizing agents. Sumoylation, the posttranslational modification with SUMO, plays an important role in the response to proteotoxic stress in the mesophilic yeast *Sc* . Therefore, we tested the possibility that perturbation of SUMO dynamics may affect *Km* ’s remarkable stress tolerance. *Km* yeast expresses variants of functionally conserved SUMO pathway genes, and we previously found that the *Km* SUMO protease KmUlp1 is considerably more tolerant to proteotoxic insults than ScUlp1 (Peek et al., 2018). Here, we tested the possibility that disruption of the non-essential SUMO protease KmUlp2, an isopeptidase related to Ulp1, may negatively affect *Km* ’s remarkable stress tolerance. To this end, we used a *Km* -specific CRISPR/Cas9 system to generate *Kmulp2* truncation mutants *in vivo* . We then compare the growth properties of these *Kmulp2* mutants and their respective WT controls to the *Sculp2* knockout mutant after exposure to various cellular stressors. Overall, we find that *Kmulp2* mutants recapitulate major phenotypes associated with the *Sculp2* mutant. This includes sensitivity to the ribonucleotide reductase inhibitor hydroxyurea and the accumulation of SUMO chains. Our study shows that KmUlp2 is required for *Km* ’s response to hydroxyurea-induced DNA damage stress. Furthermore, alterations in SUMO dynamics by Ulp2 truncations do not grossly affect the temperature tolerance of *K. m.* yeast cells.

**Figure 1. Identification and functional analysis of ULP2 SUMO protease mutants in the stress-tolerant yeast Kluyveromyces marxianus:**
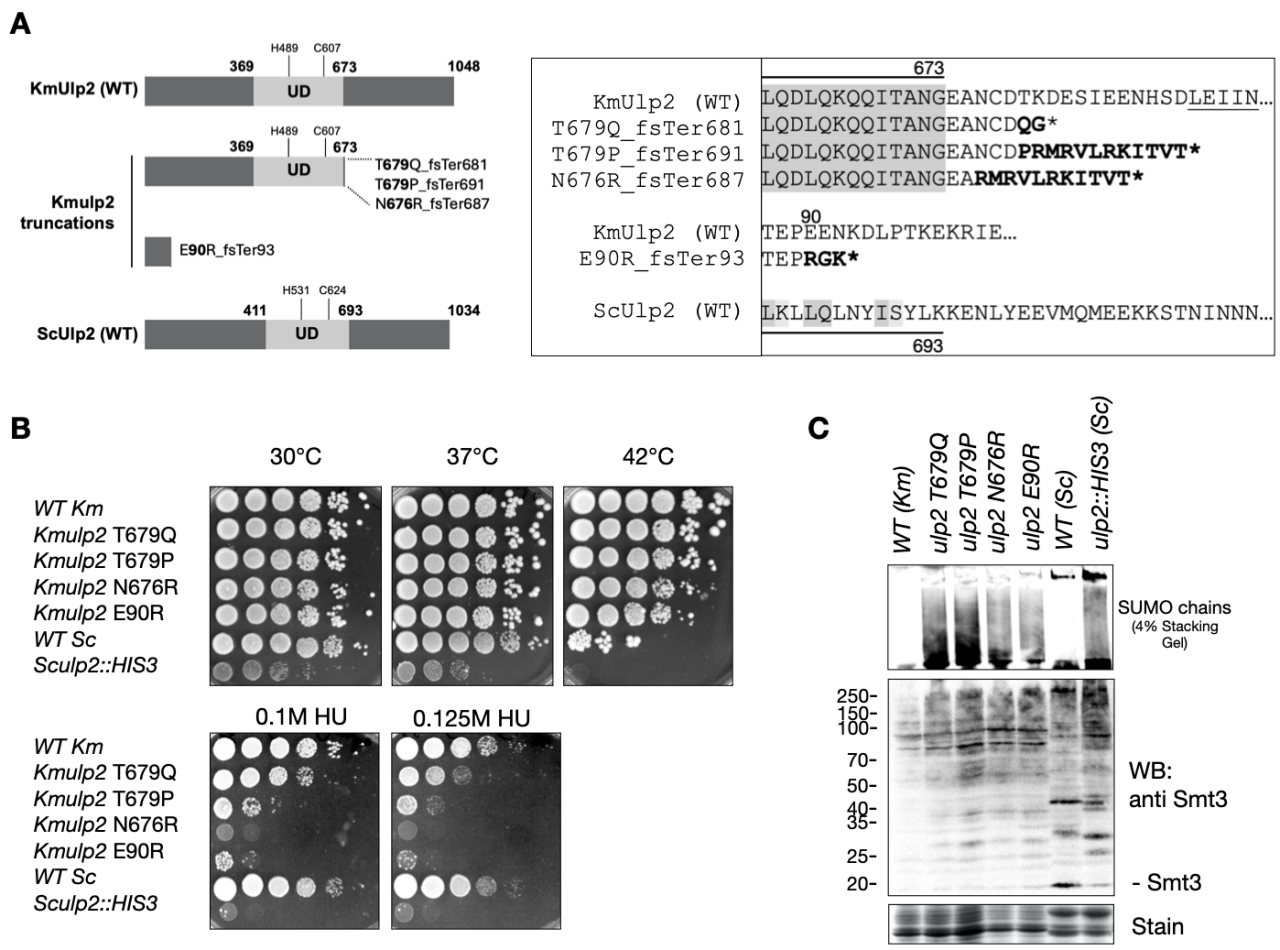
( **A** ) Left, a graphical representation of the kmUlp2 protein showing four truncation mutants of kmUlp2 created and analyzed in this study, along with ScUlp2 domains for comparison. Numbers indicate the coordinates of the catalytic UD domain, the catalytic His and Cys residues, and the length of full-length proteins. Right, amino acid sequence alignment of the indicated KmUlp2 truncation carboxy-termini with the corresponding WT Km and Sc sequence details, proximal to the catalytic UD-containing domain (highlighted in gray). Amino acids predicted to change due to the indicated frameshift mutation (fs) are shown in bold. The asterisk (*) indicates the expected position of truncation due to a termination codon. ( **B** ) Yeast cell dilution spotting assay of strains listed. Cells were 10-fold serially diluted, and 5 μl of each dilution was spotted on YPD media (top row) at the indicated temperatures, or hydroxyurea (HU) at 30°C (bottom row). Plates were incubated for 2 days (30, 37, 42°C) or 3 days (HU). ( **C** ) Western blot of whole cell extracts from the indicated strains grown at 30°C, probed with an anti-SUMO antibody. Shown are SUMO (Smt3) and sumoylated proteins in the resolving portion of the gel (anti-SUMO - middle), and SUMO chains in the stacking gel (top). A Coomassie-stained portion of the gel is shown as loading control (stain, bottom panel). Ladder markers (20-250 kDa) on the left.

## Description

Sumoylation is a conserved process that results in the reversible, covalent modification of specific eukaryotic proteins with a small ubiquitin-like protein called SUMO. The addition and removal of SUMO from a target protein involves a complex interplay of activating, conjugating, and ligating enzymes, as well as SUMO proteases (Kerscher et al., 2006). Importantly, the cellular dynamics of SUMO play a critical role in cell division, DNA repair, genome maintenance, and the response to stress and have been linked to cancer, neurodegenerative, and developmental defects (Enserink 2015; Seeler J‐S and Dejean, 2017; Ryu et al., 2020; Wang and Matunis 2023; Thu, 2024).

Extensive insights into cellular SUMO dynamics have been gained through the study of ScUlp1 and ScUlp2, two conserved yeast SUMO proteases that regulate the cellular levels of sumoylated proteins in the mesophilic budding yeast *Sc* (Hickey et al., 2012). Their substrates are mostly non-overlapping. ScUlp1 is localized to the nuclear pore complex, where it processes various substrates and the SUMO precursor, but it also gains access to sumoylated septins at the bud neck of dividing cells (Li & Hochstrasser, 2003; Elmore et al., 2011; Srikumar et al., 2013). In contrast, ScUlp2 resides in the nucleus, has fewer substrates, but efficiently processes nuclear SUMO chains (Bylebyl et al., 2003; Kroetz et al, 2009). *ScULP1* is an essential gene, and a temperature-sensitive *ulp1ts* mutant accumulates sumoylated proteins and the unprocessed SUMO precursor (Li & Hochstrasser, 1999). Deletions of the non-essential *ScULP2* gene, on the other hand, grow slowly, are temperature sensitive, accumulate SUMO chains, and are sensitive to hydroxyurea and DNA-damaging drugs (Schwartz et al., 2007; Kroetz et al, 2009; Lee et al., 2011).

The stress tolerance of *Km* yeast is positively correlated with the abundance of transcripts for ribosome assembly, translation, transcription, DNA repair, and scavenging reactive oxygen species, as well as increased thermostability for many of its proteins (Yamamoto et al., 2015; Peek et al., 2018; Yu et al., 2021). We hypothesized that altered SUMO dynamics may negatively affect the stress tolerance of *Km* . Therefore, we generated and analyzed mutations in the non-essential *ULP2* SUMO protease. To create *Kmulp2* mutants, we used a broad-host-range Cas9/gRNA expression plasmid (pUCC001) that has been used to create targeted mutations in *Km* yeast (Rajkumar et al., 2019). *KmULP2* target sequences were chosen based on the presence of an NGG protospacer adjacent motif (PAM) site in the amino and carboxy-terminal domains of *KmULP2* (nucleotide 269 and 2039). The confirmed targeting constructs were transformed into *Km* cells, and transformants were selected on YPD-Hygromycin B media plates. *Kmulp2* mutants were identified either by direct sequencing of *KmULP2* target sites of random colonies or colonies that failed to grow on 0.1M hydroxyurea. Using this approach, we identified several frameshift mutations (fs) of *KmULP2* that led to incorporation of incorrect amino acids before translation termination (Ter#), T679Q_fsTer681, T679P_fsTer691, N676R_fsTer687, and E90R_fsTer93. All truncation mutants, except E90R, retained the catalytic (UD) domain of *KmULP2* (Figure **1A** ).

Next we determined the growth phenotypes of *Kmulp2* mutations over a range of temperatures (30,37,42°C) and on media with sublethal concentrations of hydroxyurea (HU), a ribonucleotide-reductase inhibitor that causes replicative stress, recombinogenic DNA lesions, and S-phase arrest (Figure **1B** ). WT *Km* , WT *Sc* , and a *Sculp2::HIS3* mutant were used as controls and comparators. This analysis revealed that, unlike the *Sculp2::HIS3* deletion mutant, *Kmulp2* mutants did not exhibit a slow growth phenotype at 30°C or temperature sensitivity at 37°C. All *Km* WT and *ulp2* mutant strains, even kmE90R that retains only the first 90 amino acids of kmUlp2, grew robustly up to 42°C. Our sensitive dilution spotting assay revealed a mild growth delay at 42°C for the N676R mutant and the E90R mutant, resulting in smaller colonies. As expected, *Sc* WT and *ulp2::HIS3* deletions grew poorly or not at all at 42°C, and as previously reported, *Sc ulp2::HIS3* was temperature-sensitive and failed to grow at 37°C (Li and Hochstrasser, 2000; Kroetz et al, 2013). In contrast, all *Km* truncations, no matter if they retained the catalytic core domain or not, showed severe growth defects at 0.1µM and 0.125µM HU, as did the *Sc ulp2::HIS3* control. (Figure **1B** - lower panel). One exception was the T679Q mutant, which was slightly more resistant to HU, but still did not match the robust growth of the *Km* and *Sc* WT strains. These data reveal that both *Km* and *Sc* *ULP2* mutants are sensitive to HU-induced replicative stress, but *Kmulp2* truncations tested by us remain resistant to heat stress and do not exhibit a slow growth phenotype.

Considering the clear difference between the temperature-sensitive growth properties of *Kmulp2* truncations and the *Sculp2::HIS3* deletion, we decided to investigate the formation of SUMO chains in these strains. We extracted proteins from our *Km* mutants, the *Sc* mutant, and WT strains and used an antibody that detects SUMO and SUMO chains in both *Km* and *Sc* cells after SDS-PAGE gel electrophoresis and western blotting. One hallmark of *Sculp2* mutants is the accumulation of unprocessed SUMO chains in the stacking gel of SDS-PAGE gels that can be detected after western blotting (Bylebyl et al., 2003; Schnellhardt et al., 2012; Kroetz et al; 2009). Comparing *Sculp2* and *Kmulp2* mutants after growth at 30°C, we were able to show that SUMO chains accumulate in *Km* cells (Figure **1C** ). As expected, SUMO chains did not accumulate in WT *Km* and the *Sc* control. These data suggest that *kmulp2* truncations, whether they retained the catalytic core domain or not, are functionally defective in the ability to process SUMO chains.

In summary, we show that mutations that truncate the SUMO protease *ULP2* in the stress-tolerant *Km* yeast recapitulate select phenotypes associated with *ULP2* mutations in *Sc* . This includes HU-sensitivity and SUMO-chain accumulation, which have previously been observed in *Sculp2∆C* mutants that are structurally similar to *kmT679Q* , *kmT679P* , and *kmN676R* truncations (Kroetz et al, 2009). In addition to these phenotypes, strains with complete deletions *of ScULP2* and deletions of the N-terminus ( *Sculp2∆N* ) grow poorly under all growth conditions and are also temperature sensitive (Kroetz et al., 2009). However, the kmE90R mutant, which lacks all but the first 90 amino acids of kmUlp2, may functionally resemble a knockout and retains the ability to grow robustly at 37 and 42°C. This could mean that heat tolerance in Km yeast is not highly dependent on having functional Ulp2 isopeptidase activity. Though we cannot exclude the possibility that a complete deletion of *kmULP2* is also heat sensitive, our data suggest that alteration in SUMO dynamics by *kmulp2* truncations (including *kmE90R* ) does not significantly affect temperature tolerance in these mutants.

While the basis for the slow growth temperature sensitivity of *Sculp2∆* cells is not clear, one possible explanation is that in *Km* yeast the lack of Ulp2 is overcome by other means. For example, we hypothesize that the SUMO protease KmUlp1 can partially complement severe *kmulp2* mutations like *kmE90R* . Indeed, we previously showed that, unlike ScUlp1, kmUlp1 remains active even under adverse conditions such as 42°C and other proteotoxic insults (Peek et al., 2018). However, how KmUlp1 or other proteins may suppress any growth defects of *Kmulp2* mutants is currently not clear.

The observation that all *Kmulp2* mutants, regardless of retaining the catalytic core domain, were HU-sensitive is not entirely unexpected. Kroetz and co-workers previously found that a *Sculp2∆C* mutant that lacks the c-terminal domain (CTD) of ScUlp2 past amino acid 693, but retains the catalytic UD domain, was HU-sensitive and contained elevated levels of high-molecular-mass poly-SUMO chains (Kroetz et al., 2009). It was also reported that a SUMO-interacting motif (SIM) in the CTD, and possible other features in this domain, are involved in recruiting substrates for Ulp2 (Kroetz et al., 2009; Albuquerque et al., 2018 **)** . Correspondingly, KmUlp2 has at least three putative SIMs that reside in an unstructured CTD (690LEIIE694, 951ISLEL955, and 991VEEIL995) and are absent in our *Km* truncations (Guo et al., 2024). The inability to interact with SUMO may explain why all *kmulp2* mutants tested accumulate SUMO chains. Presently, we are unable to discern a clear correlation between the HU-sensitivity of our *km* mutants and the level of SUMO chain accumulation. Therefore, as a caveat to this interpretation, it is not entirely clear why *Kmulp2* T679Q grows slightly better than the other mutants on HU-containing media.

Overall, our study shows that using a *Km* -adapted CRISPR system is a useful means to investigate the role that SUMO dynamics play in the remarkable stress tolerance of this organism.

## Methods

Generation and analysis of *Kmulp2* mutants: *KmULP2* target sequences were selected using Benchling (www.benchling.com) based on the presence of an NGG protospacer adjacent motif (PAM) site in the amino and carboxy-terminal domains of kmULP2 (nucleotide 269 and 2039 of *kmULP2* ). Corresponding 20-basepair Cas9 gRNA target sequences (see Materials and Methods) were ordered as single-stranded primers ( www.idtdna.com ), annealed, and cloned into Bsa1-cut pUCC001 (addgene Plasmid #124451, PMID: 31134195; BsaI-HF®v2 enzyme, NEB R3733S). Transformation into *Km* cells was accomplished using a high-efficiency yeast transformation protocol with LiSORB and LiPEG, based on the High-Efficiency Yeast Plasmid Transformation from Stan Field’s lab. Standard YPD-media with 200µg/ml hygromycin B (USBiological, H9700-05B) was used to select colonies containing the targeting constructs. Overall, recovery of colonies with successful *kmULP2* disruptions was low (ranging from 2-20% based on targeting constructs), and so we pre-screened *Km* colonies for sensitivity to hydroxyurea (USBiological, H9120) and/or used colony PCR to identify correctly targeted mutants. Functional assays with individual mutants were performed at the indicated temperatures, using 0.1M or 0.125M hydroxyurea. SDS-PAGE gel electrophoresis was performed using large, 1mm thick, homemade 10% acrylamide/bis gels with 4% stacker. Running and western blotting conditions were as described (Schnellhardt et al., 2012) except that 4 OD600 worth of whole cell extracts were used per lane. Blocking and antibody incubations were performed in blocking buffer (Everyblot, Biorad #12010020) with a polyclonal anti-SUMO antibody (a gift from the lab of Mike Matunis). To visualize SUMO chains in the stacker, primary antibody incubation with the anti-SUMO antibody was performed at 1:5000 dilution overnight at 4°C. After washing steps and incubation with the secondary antibody (Cell Signaling #7074), blots were imaged using a Thermo Fisher iBright Imaging system.

## Reagents

Oligonucleotides for guide RNAs:

**Table:** 

OOK 1394	ULP2Km_pUCC001_FWD	Used to target nucleotide position 2039	CGTCTGAAGCTAACTGCGACACCA
OOK 1395	ULP2Km_pUCC001_REV	Used to target nucleotide position 2039	AAACTGGTGTCGCAGTTAGCTTCA
OOK 1472	811380_gRNA_fwd_KmULP2	Used to target nucleotide position 269	CGTCGATTGATATTACCGAACCAG
OOK 1473	811380_gRNA_rev_KmULP2	Used to target nucleotide position 269	AAACCTGGTTCGGTAATATCAATC

Cas9/gRNA expression plasmids:

**Table:** 

BOK 1801	pUCC001_ULP2 targeting construct used to create T679Q, T679P, N676R mutations in *KmULP2*
BOK 1930	pUCC001_ULP2 targeting construct used to create E90R mutation in *KmULP2*

Yeast Strains:

**Table:** 

BY28353	*ura3-1 ade2-2 leu2-2 (Kluyveromyces marxianus DMKU3-1042)*	Yarimizu et al., 2013
YOK4382	*kmulp2_* T679Q_fsTer681 in *Km* strain BY28353	This work
YOK4506	*kmulp2_* T679P_fsTer691 in *Km* strain BY28353	This work
YOK4603	*kmulp2_* N676R_fsTer687 in *Km* strain BY28353	This work
YOK4633	*kmulp2_* E90R_fsTer93 in *Km* strain BY28353	This work
MHY500	*Matα his3-Δ200, leu2-3, 112, ura3-52, lys2-801, trp1-1 gal2*	Li and Hochstrasser, 2003
DSY858	*Mata ade2-1, his3, leu2-3, 112, trp1-1, ura3-52, ulp2::HIS3*	a gift from Mark Hochstrasser
